# A Cross-Sectional Study of the Use of Antigen Rapid Diagnostic Tests for Community Identification of SARS-CoV-2 in Kenya

**DOI:** 10.4269/ajtmh.23-0756

**Published:** 2024-11-26

**Authors:** Rose Masaba, Stephen Siamba, Heather J. Hoffman, Edyth Osire, Njoki Kimani, Magoma Kwasa, Mario Songane, Lise Denoeud-Ndam

**Affiliations:** ^1^ Elizabeth Glaser Pediatric AIDS Foundation, Nairobi, Kenya;; ^2^ The George Washington University, Washington, District of Columbia;; ^3^ Kiambu County Government, Ministry of Health, Kiambu, Kenya;; ^4^ Elizabeth Glaser Pediatric AIDS Foundation, Maputo, Mozambique;; ^5^ Elizabeth Glaser Pediatric AIDS Foundation, Geneva, Switzerland

## Abstract

Mass testing with antigen-detecting rapid diagnostic tests (Ag-RDT), including testing of asymptomatic individuals, is expected to improve the identification of severe acute respiratory syndrome coronavirus-2 (SARS-CoV-2) infections. Mass testing was offered at large gatherings to determine the SARS-CoV-2 case detection rate and the acceptance and cost of implementing this community testing strategy. In 49 high-attendance venues in Kiambu County, Kenya, from June to September 2022, individuals 2 years and older were offered coronavirus disease 2019 (COVID-19) testing, vaccination, and participation in a survey. Polymerase chain reaction (PCR) testing and genome sequencing were conducted for those testing positive by Ag-RDT and those testing negative but with COVID-19 symptoms. Costs were collected from financial records, budgets, and invoices and estimated from a health systems perspective using a micro-costing method. A total of 4,062 individuals were offered testing. The testing acceptance was 3,174/4,062 (78.1%). The case detection rate was 34/3,174 (1.07%; 95% CI: 0.7–1.4%), and 11/34 (32%) of the positives were asymptomatic. The PCR results were available for 27 Ag-RDT‒positive participants and 14 Ag-RDT‒negative participants with SARS-CoV-2 symptoms and were positive in 24/27 (88.9%) and 4/14 (28.6%), respectively. Circulating variants were identified in 11 participants. Community mobilization was the major cost driver (26%) followed by purchase of SARS-CoV-2 Ag-RDTs (20.5%). The cost per individual tested was USD $15.89, and the cost per individual tested positive for SARS-CoV-2 was USD $1,484. The study demonstrates that SARS-CoV-2 Ag-RDTs could be used for identification of SARS-CoV-2 infections in both symptomatic and asymptomatic individuals at mass gatherings.

## INTRODUCTION

Since the identification of the first case of severe acute respiratory syndrome coronavirus 2 (SARS-CoV-2), the cause of the respiratory disease known as coronavirus disease 2019 (COVID-19), in December 2019 in China, the number of cases has exponentially increased globally to reach 775,867,547 confirmed cases and over 7 million related deaths worldwide as of August 2024.^1^ In Africa, 9.5 million SARS-CoV-2‒confirmed cases and 175,526 related deaths were reported for the same period, although this likely represents a significant underestimation given the limited access to SARS-CoV-2 testing.^1^ The WHO has noted that African countries are underreporting SARS-CoV-2 case burden owing to low testing rates; as many as six out of seven cases of infection are not detected.^2^

The pandemic affected the health systems of African countries and the management of major preexisting infectious diseases such as HIV and tuberculosis.^3^

The SARS-CoV-2 intervention tools, including vaccines, testing, and therapeutics, were quickly developed, but availability, access, and uptake are still limited in most African nations. National economic constraints, poverty, low health literacy rates, and poor risk communication have further impeded disease control.^4^

In addition, it is essential to identify asymptomatic individuals with SARS-CoV-2 infection at the community level, as this can be a major driver of transmission of infection, as has been reported in previous studies. A decision analytical model based on eight studies estimated that 59% of SARS-CoV-2 transmissions occurred from asymptomatic cases.^5^ In a systematic review including 16 studies, the prevalence of asymptomatic individuals was 48.2% from a total of 2,788 confirmed cases of SARS-CoV-2 infection.^6^ Another systematic review aiming to estimate the proportion of persons infected with SARS-CoV-2 who never developed symptoms found that nearly three-quarters of persons who received a positive PCR test result but had no symptoms at the time of testing remained asymptomatic. It concluded that control strategies for SARS-CoV-2 should be altered, considering the prevalence and transmission risk of asymptomatic SARS-CoV-2 infection.^7^

As of October 2022, 35.8% of adults had received a SARS-CoV-2 vaccine in Kenya, defined as having received at least one dose of SARS-CoV-2 vaccine but not necessarily the full vaccination series.^8^ Until vaccines have widespread availability, testing and isolation are the best methods to control infection spread at the national level. The use of simple, rapid, and affordable antigen-detecting rapid diagnostic tests (Ag-RDTs) to expand access to SARS-CoV-2 testing is being incorporated in many national pandemic responses, especially in low- and middle-income countries (LMICs).^9^^,^^10^ Targeting high-volume community venues has the potential to screen many people in a limited amount of time and determine any geographic areas with elevated community transmission. Transmission in high-volume venues such as music clubs, churches, conference facilities, and nursing facilities has been reported in previous studies, in Osaka, Japan, and Boston, MA.^11^^,^^12^

Testing with Ag-RDTs, including testing of asymptomatic individuals, has the potential to identify more SARS-CoV-2 infections and consequently decrease the spread of infection at the community level.^13^ In addition, rapid test results are important for immediate clinical management and isolation of patients with SARS-CoV-2 infection and for contact tracing and quarantining of contacts.^14^ Data on SARS-CoV-2 infection rates, the acceptability of Ag-RDT, and the cost of conducting widespread testing in high-volume venues are limited in Africa.

Most testing programs in LMICs use a screen-and-test strategy to identify symptomatic infection and those at risk as a result of known exposure because of the limited availability and costs of broader, more universal testing.^15^ However, this strategy does not identify those with asymptomatic infection who also contribute to the spread of SARS-CoV-2 infection. Data are needed to inform the evolving SARS-CoV-2 testing guidelines and provide the Ministry of Health (MOH) with costed testing models to complement its national strategies. This study aimed to 1) determine the SARS-CoV-2 case detection rate using Ag-RDTs across selected high-volume community testing venues, 2) determine the proportion of asymptomatic and symptomatic infections detected using SARS-CoV-2 Ag-RDTs in these testing venues, 3) identify factors associated with SARS-CoV-2 infection, 4) determine the cost of implementing a community testing strategy in selected high-volume community testing venues, and 5) characterize circulating SARS-CoV-2 variants among those with a positive polymerase chain reaction (PCR) test in these venues.

## MATERIALS AND METHODS

### Study design, population, and sites.

We conducted a cross-sectional study targeting approximately 5,000 persons and offered testing in 49 high-attendance venues in Kiambu County that were identified as possible points of community-based transmission. The sample of 5,000 was a convenience sample based on the available budget, time, and sites that were selected for conducting the study.

The study was conducted from June to September 2022 in markets, stadiums, bus parks, shopping centers, recreational parks, and chief’s camps (local administrative offices) in 12 sub-counties in Kiambu County, Kenya. Kiambu County neighbors the capital city, Nairobi, and ranks second in the country in cumulative number of confirmed SARS-CoV-2 cases.^16^ The county had major gaps in SARS-CoV-2 management owing to inadequate resources limiting response capacity in detection, investigation, contact tracing, and follow-up within both healthcare facilities and the community. The county reported spikes in SARS-CoV-2 cases after participation in mass gathering events such as political rallies, market visits, church services, and funerals and at tertiary institutions. Venues were identified in collaboration with the Department of Public Health and the COVID-19 response team in the county, based on available data on areas that were considered transmission hotspots. Each sub-county provided at least four high-volume venues for inclusion in the study.

The study population was composed of all persons visiting a selected high-volume community venue that had been identified as a possible point of community-based transmission on the day of the SARS-CoV-2 testing campaign. Children aged >2 years and adults of all ages were included, regardless of presence or absence of COVID-19 symptoms. Individuals were eligible to participate in the study if they agreed to undergo testing with a SARS-CoV-2 Ag-RDT on the day of the testing campaign and if, after receiving information about the testing, they were able and willing to provide written informed consent/assent for both testing and a post-test interview. We excluded individuals who reported a positive SARS-CoV-2 test result within 14 days of the current testing campaign. After providing written informed consent, enrolled participants were screened for symptoms and referred to the testing team on-site. Those who had no symptoms and tested negative for SARS-CoV-2 using the Ag-RDT were considered uninfected and offered SARS-CoV-2 vaccination. Those who had COVID-19 symptoms and tested negative and those who tested positive on a SARS-CoV-2 Ag-RDT were asked for consent to provide a second sample for PCR testing and subsequent genomic sequencing.

### Community mobilization.

To ensure a high response rate and successful study implementation, the Kiambu County COVID-19 response team was engaged from the initial stages of the testing campaign to mobilize the community. Before the mass testing, meetings were held with community leaders to explain the study and to discuss potential problems and concerns, including preventing dissemination of false information. These meetings were coordinated by the county public health department in close collaboration with the study team. Key stakeholders included social workers; community health volunteers; local community leaders (chiefs, sub-chiefs); members of the county and sub-county health management teams, including those from the public health department; the county COVID-19 response manager; the county education department; school principals; and religious leaders, among others. A few days before the start of the testing campaign, community health volunteers visited the selected high-attendance venues and distributed a poster announcing the community testing activity for display in the venue prior to the scheduled testing intervention. Trained community health volunteers and mobilizers also visited the venue on agreed upon days to announce the testing day and mobilize the community. Available channels such as community WhatsApp groups, Facebook, and other social media platforms were also used to mobilize the community. Through church leaders and pastors’ associations, messages about the testing were posted on church websites, where available. Cars mounted with megaphones were also used for community mobilization on the testing day.

### Testing procedures.

Testing for SARS-CoV-2 was conducted by trained MOH laboratory technologists using WHO- and country-approved tests. All SARS-CoV-2 sample collection and testing procedures were conducted according to national and manufacturer guidelines.

Nasopharyngeal swabs were obtained from consenting participants, and rapid antigen tests were conducted using the Abbott Panbio (Jena, Germany) test in the first 28 venues and the SD Biosensor test (Suwon, Republic of Korea) in the last 21 venues. A second nasopharyngeal swab for PCR testing and subsequent genome sequencing was collected from all participants who tested positive by Ag-RDT and from those who tested negative by Ag-RDT but had symptoms consistent with COVID-19. The nasopharyngeal swabs were collected after obtaining a second written informed consent form. The swabs were immersed in viral transport media, stored between –20°C and –80°C, and shipped to the testing laboratory where PCR testing and subsequent genome sequencing were conducted.

### Data collection and storage.

Qualified research staff with experience conducting surveys were hired and trained for 5 days, including pilot testing of the data collection tools. Using a participatory approach, the training provided theoretical background and discussion of study procedures and provided guidance on quantitative data collection and review of all data collection tools and relevant standard operating procedures. Role plays were conducted to ensure that the staff fully understood the tools. Research staff received ethics training to ensure compliance with human subject research requirements and signed a confidentiality agreement before engaging in study activities. The study data collection tools were adapted from the screening forms developed by the WHO^17^ and adapted by the MOH and did not contain any patient identifiers. Data collected included demographic characteristics and SARS-CoV-2 infection history, including vaccination. In addition to the study tools, the MOH Ag-RDT laboratory register was completed as part of routine COVID-19 response data collection by the sub-county MOH. Details in the register included patient identifiers for patient management and reporting.

Data collection tools were completed at the testing venue with data entered directly into an electronic form installed on tablets carried by research staff. Data were transferred into an electronic database using the ODK-X application, designed specifically for the study, and stored on a secure web-based server. The data collection tool had built-in data checks to ensure that collected data were within range and that related questions had logical responses. Out-of-range values were verified before transmission to the electronic database through a data query process implemented by the data manager. In testing venues that did not have internet connectivity suitable for the immediate uploading of data, data were stored temporarily on the tablet and subsequently transferred in an encrypted format to the web-based server. The data on the server were backed up every 24 hours, and the tablets were synced to a computer daily. All electronic tablets used in collecting data were password protected and accessible only to key study personnel. Patient data were only identified using a unique study ID issued at enrollment into the study. Access to the database was controlled through receipt of an individual password-protected log-in requirement with differential levels of access as needed for study staff and investigators. We performed secondary cost data collection from project financial records, budgets, and invoices. Ministry of Health personnel costs were received directly from the MOH.

## STATISTICAL ANALYSES

We described the distribution of demographic characteristics of the study participants using frequencies and proportions for categorical variables and means and SDs or median and interquartile (IQR) ranges for continuous variables, as appropriate. Study participant characterization was further disaggregated by community testing venue. We estimated both the proportion of people accepting testing and the proportion of SARS-CoV-2‒positive tests among tested participants (overall and in relevant subgroups), with binomial exact CIs around the estimate. Logistic regression modeling was used to determine factors associated with SARS-CoV-2 infection. Odds ratios and associated 95% CI were estimated. Factors with CI excluding 1 were considered significantly associated with infection. Frequencies and proportions of SARS-CoV-2 variants were estimated among SARS-CoV-2 PCR-positive individuals. Statistical analysis was conducted using STATA Statistical Software v. 16.0 (College Station, TX).

### Cost analysis.

Costs were estimated from a health systems perspective using a micro-costing method, combining top-down and bottom-up approaches to obtain resource use and costs per line item. All project costs were converted to 2022 U.S. dollars (USD $) using the prevailing exchange rate from the Central Bank of Kenya. Costs were divided into the following categories: personnel, supplies (including SARS-CoV-2 Ag-RDTs), equipment, travel, community mobilization, and meetings. For personnel, staff costs were calculated using the level of effort each cadre or employee dedicated to this project, their monthly salary, and the number of months worked on the project. For MOH staff, the costs were calculated by multiplying the total number of days they worked and their daily salary. Equipment (laptops) was treated as a capital cost using a useful life of 5 years (www.statista.com) and a discount rate of 3% according to WHO guidelines,^18^ and costs were annualized by dividing the total cost of equipment by the annuity, as previously done by Walker and Kumaranayake^19^ and Kimaro et al.^20^

To obtain cost estimates per individual tested for SARS-CoV-2 and per individual testing positive for SARS-CoV-2, we calculated the total cost of implementing community testing and then divided it by the number of individuals tested for SARS-CoV-2 and the number of individuals testing positive for SARS-CoV-2, respectively. Our cost estimation methodology was modeled upon that of Mwenge et al.^21^ and Vyas et al.^22^

## RESULTS

From June to September 2022, 49 different venues were visited in the 12 sub-counties of Kiambu County during 12 weeks of community testing (Table 1). Testing venues included 31 markets or shopping centers, five chiefs’ camps (administrative offices), six bus parks, two stadiums, and five other venues (a railway station, a prison, a church, a town center, and a recreational park).

**Table 1:** Screening, enrollment, and Ag-RDT testing in 12 sub-counties of Kiambu County, Kenya, June‒September 2022 (chronologically ordered)

Sub-County	Testing Week	Number of Testing Days (number of venues)*	Screened for Study Eligibility	Enrolled (%)
Limuru	June 20	4 (4)	244	187 (76.6)
Kabete	June 27	4 (4)	352	318 (90.3)
Lari	July 4	4 (4)	326	254 (77.9)
Gatundu N.	July 11	4 (4)	274	165 (60.2)
Gatundu S.	July 18	4 (4)	284	196 (69.0)
Kikuyu	July 25	4 (4)	453	355 (78.4)
Kiambaa	August 1*	4 (4)	372	315 (84.7)
Kiambu	August 29	4 (4)	464	392 (84.5)
Ruiru	September 5	5 (4)^†^	529	498 (94.1)
Thika Town	September 12	3 (3)	128	77 (60.2)
Githunguri	September 19	5 (5)	321	275 (85.7)
Juja	September 26	5 (5)	315	258 (81.9)
Total	‒	50 (49)	4,062	3,290 (81.0)

Ag-RDT = antigen-detecting rapid diagnostic test.

* There was a 3-week pause in testing activities during the August general elections.

^†^ Each day, a different venue was visited in the same sub-county. The only exception is in Ruiru sub-county, where one venue was visited twice, on Monday and Friday.

Table 1 shows the sub-counties where Ag-RDT testing was conducted on different dates from June to September 2022. Those screened for study eligibility and enrolled into the study and the number of testing days in each sub-county were captured. There was a 3-week pause in testing activities during general elections in August 2022.

**Figure 1.**
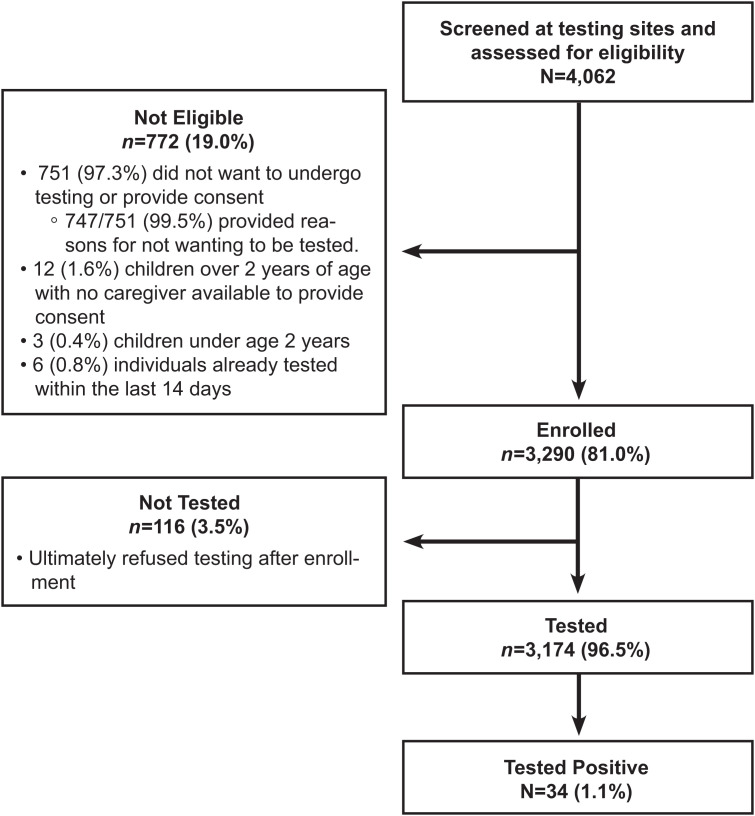
Diagram of participants who attended the WT for COVID-19 screening using Ag-RDTs.

A total of 4,062 individuals were screened for eligibility, of whom 3,290 (81%) were enrolled in the study (Figure 1). Among the 772/4,062 (19.0%) who did not meet study eligibility criteria, the reasons for ineligibility were distributed as follows: 751/4,062 (18.5%) did not want to undergo testing or to provide consent; 12 (0.3%) were children over the age of 2 years without a parent or caregiver to provide consent; three were children under 2 years (0.07%); and (0.15%) had already been tested within the last 14 days. The primary reasons why individuals seen at the testing venues stated they did not want to be tested were that they saw no value in or no need for the test and thought they were healthy; they disliked or felt uncomfortable with the testing procedure; and they feared the test (either fear of pain from the conduct of the test itself or fear of the result).

A total of 3,174/4,062 (78.1%) participants were tested for SARS-CoV-2 using the Ag-RDT, whereas 116 of the 3,290 (3.5%) enrolled participants decided after enrollment that they did not want the SARS-CoV-2 test (Figure 1).

### Characteristics of enrolled participants disaggregated by their testing result status.

Table 2 represents the characteristics of participants enrolled in the study. Overall, 2,114/3,290 (64.3%) were male; the median age was 39 (IQR: 27‒53) years. About half of the enrolled participants were age 40 years or above (1,633/3,290, 49.6%); 63.1% of the participants had received a SARS-CoV-2 vaccine. The majority (98.1%) of the participants did not have a history of SARS-CoV-2 infection in the past. Overall, 2,970/3,290 (90.3%) of the enrolled participants did not report any COVID-19 symptoms. Among people who tested Ag-RDT negative or with testing not done, 297/3,256 (9.1%) had symptoms consistent with potential COVID-19, whereas among people who tested Ag-RDT positive, 23/34 (67.6%) had symptoms. The most common symptoms reported by the SARS-CoV-2 Ag-RDT‒positive participants were runny nose (61.8%), cough (47.1%), headache and muscle aches (32.4%), and sore throat (23.5%). About half of those who had symptoms had sought clinical care in the 10 days prior to testing.

**Table 2:** Characteristics of participants enrolled in the study by Ag-RDT testing result

Factor	Level	Positive *n* = 34	Negative *n* = 3,140	Not done *n* = 116	Total (*N* = 3,290)
Sex	Male	20 (58.8%)	2,026 (64.5%)	68 (58.6%)	2,114 (64.3%)
	Female	14 (41.2%)	1,114 (35.5%)	48 (41.4%)	1,176 (35.7%)
Age (years)	Median (IQR)	49 (32‒64)	39 (28‒53)	35 (22‒45)	39 (27‒53)
	2–18	2 (5.9%)	146 (4.6%)	18 (15.5%)	166 (5.0%)
	19–29	4 (11.8%)	761 (24.2%)	30 (25.9%)	795 (24.2%)
	30–39	5 (14.7%)	671 (21.4%)	20 (17.2%)	696 (21.2%)
	40+	23 (67.6%)	1,562 (49.8%)	48 (41.4%)	1,633 (49.6%)
Vaccinated for SARS-CoV-2*	Yes	22 (64.7%)	1,991 (63.4%)	64 (55.2%)	2,077 (63.1%)
Number of SARS-CoV-2 Vaccine Doses Received^†^	1 Dose	3 (8.8%)	483 (15.4%)	24 (20.7%)	510 (15.5%)
	2 Doses	17 (50.0%)	1,225 (39.0%)	35 (30.2%)	1,277 (38.8%)
	3 Doses	2 (5.9%)	283 (9.0%)	5 (4.3%)	290 (8.8%)
Ever Diagnosed with SARS-CoV-2?	Yes	0 (0.0%)	61 (1.9%)	1 (0.9%)	62 (1.9%)
Traveled Out of Kenya in the Past 2 Weeks?	Yes	0 (0.0%)	36 (1.1%)	2 (1.7%)	38 (1.2%)
Contact with a Confirmed or Suspected SARS-CoV-2 Case in the Last 7 Days?	Yes	1 (2.9%)	74 (2.4%)	2 (1.7%)	77 (2.3%)
Has Had COVID-19 Symptoms in the Last 10 Days?	Yes	23 (67.6%)	288 (9.2%)	9 (7.8%)	320 (9.7%)
Most Common COVID-19 Symptoms in the Last 10 Days?	Runny Nose	21 (61.8%)	189 (6.0%)	6 (5.2%)	216 (6.6%)
	Cough	16 (47.1%)	186 (5.9%)	7 (6.0%)	209 (6.4%)
	Headache	11 (32.4%)	105 (3.3%)	3 (2.6%)	119 (3.6%)
	Muscle Aches	11 (32.4%)	49 (1.6%)	1 (0.9%)	61 (1.9%)
	Fever	7 (20.6%)	70 (2.2%)	4 (3.4%)	81 (2.5%)
	Sore Throat	8 (23.5%)	75 (2.4%)	3 (2.6%)	86 (2.6%)
If Symptoms Present, Sought Any Medical Care in Last 10 Days?	Yes	10 (43.5%)	140 (48.6%)	4 (44.4%)	154 (48.1%)

Ag-RDT = antigen-detecting rapid diagnostic test; COVID-19 = coronavirus disease 2019; IQR = interquartile range; SARS-CoV-2 = severe acute respiratory syndrome coronavirus-2.

* Yes implies they had received at least one dose of SARS-CoV-2 vaccine but not necessarily the full vaccination series.

^†^ Applicable for those who had been vaccinated.

Table 2 represents both demographic and clinical characteristics of participants enrolled in the study by Ag-RDT results. Results are shown as either positive or negative and for those who did not undertake the test.

### Case detection rate.

Of the 3,174 participants who were tested for SARS-CoV-2 using an Ag-RDT, 34 were diagnosed with SARS-CoV-2 infection, giving a case detection rate of 34/3,174 (1.07%; 95% CI: 0.7–1.4%). Eleven of the 34 who tested positive for SARS-CoV-2 using an Ag-RDT (32%) were asymptomatic.

Table 3 shows the testing coverage, acceptability, and case detection rate by venue type. The average proportion of participants tested each day was 81.2; it reached 83.6 in markets and shopping centers. The overall case detection rate was 1.2% (95% CI: 0.8–1.8%) for markets/shopping centers, 0.8% (95% CI: 0.1–3.0%) for chief’s camps, and 2.7% (95% CI: 1.9–5.3%) for bus parks.

**Table 3:** Testing coverage, acceptability, and case detection rates in the different venues

Venue Type (number of days)	Number Offered Testing (average per testing day)	Number Tested (average per day)	Percentage Tested among Those Offered Testing* (95% CI)	Number of Ag-RDT Positive (average per day)	Percentage of Ag-RDT‒Positive Tests among Tested^†^ (95% CI)
Market/Shopping Center (*n* = 31)	2,591 (83.6)	1,998 (64.5)	77.1 (75.4–78.7)	24 (0.8)	1.2 (0.8–1.8)
Chief’s Camp (*n* = 5)	328 (65.6)	242 (48.4)	73.8 (68.7–78.5)	2 (0.4)	0.8 (0.1–3.0)
Bus Parks (*n* = 6)	397 (66.2)	293 (48.8)	73.8 (69.2–78.1)	8 (1.3)	2.7 (1.9–5.3)
Stadium (*n* = 2)	148 (74)	109 (54.5)	73.6 (65.8–80.5)	0 (0)	0
Others (*n* = 5 venues, 6 days)^‡^	598 (99.7)	532 (88.7)	89.0 (86.2–91.4)	0	0
Overall (*N* = 50)	4,062 (81.2)	3,174 (63.5)	78.1 (76.8–79.4)	34 (0.7)	1.07 (0.7–1.4)

Ag-RDT = antigen-detecting rapid diagnostic test.

* *P*-Value for the comparison between venue types is <0.0001, Fisher exact test.

^†^ *P*-value for the comparison between venue types is 0.003, Fisher exact test.

^‡^ Other venues included are one railway station (visited twice), one prison, one church, one town center, and one recreational park. The “others” category accounts for five venues and six testing days.

Table 3 shows the number and proportions offered testing and those accepting testing on average per day of testing by venue type. It also shows the number and proportions of those testing positive per day of testing.

### Factors associated with SARS-CoV-2 infections as detected by rapid diagnostic tests.

There were no significant associations between SARS-CoV-2 infection and factors such as sex, venue type, exposure history, and vaccination status (Table 4). A nonsignificant trend was observed of higher odds of positive Ag-RDTs in younger (2–18 years) and older (>40 years) age categories, whereas the 19–39-year age group appeared to have the lowest infection rate (*P* = 0.19).

**Table 4:** Logistic regression analysis of factors associated with SARS-CoV-2 infection

Factor	Crude OR (95% CI)	*P*-Value	Adjusted OR [95% CI]	*P*-Value
Sex				
Male	1	0.491	‒	–
Female	1.3 (0.6–2.5)		‒	
Age (years)				
2–18	1	0.186	1	0.902
19–29	0.4 (0.1–2.1)		0.8 (0.1–5.0)	
30–39	0.5 (0.1–2.8)		1.2 (0.2–6.9)	
40+	1.1 (0.4–4.6)		1.3 (0.3–5.8)	
Venue Type				
Market/Shopping Center	1.4 (0.7–3.0)	0.356	‒	‒
Other	1		‒	
Contact with a Confirmed/Suspected SARS-CoV-2 Case in the Last 7 Days				
No	1	0.673	‒	‒
Yes	1.3 (0.2–10.0)		‒	
Unknown	1.5 (0.6–3.6)		‒	
COVID-19 Symptoms in Last 10 Days				
No	1	<0.001	1	<0.001
Yes	20.7 (10.0–42.9)		12.8 (6.1–27.1)	
SARS-CoV-2 Vaccinated				
No	1	0.876	‒	–
Yes (at least one dose)	1.1 (0.5–2.1)		‒	
Period of Ag-RDT Test				
Weeks 1–4 of Testing (June 20‒July 15) (>100 new cases per day country-wide)	1	<0.0001	1	0.001
Weeks 5–8 of Testing (July 18‒Sept. 1) (10–100 new cases per day country-wide)	0.05 (0.01–0.20)		0.1 (0.02–0.3)	
Weeks 9–12 of Testing (Sept. 5‒30) (<10 new cases per day country-wide)	0.04 (0.02–0.05)		0.2 (0.01–3.6)	
Ag-RDT Type				
SD Biosensor	4	<0.0001	1	0.164
Panbio	15.1 (3.6–63.1)		4.2 (0.6–32.0)	

Ag-RDT = antigen-detecting rapid diagnostic test; COVID-19 = coronavirus disease 2019; OR = odds ratio; SARS-CoV-2 = severe acute respiratory syndrome coronavirus-2. The association of test positivity with variables of interest was first tested in univariable models. The multivariable model included variables at a significance of 0.20 in univariable models.

**Figure 2.**
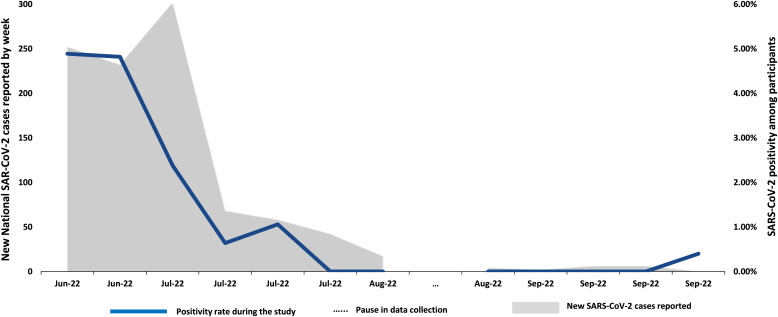
Comparison of study SARS-CoV-2 positivity rate and national SARS-CoV-2 cases reported during the data collection period, June 20‒September 26, 2022. John Hopkins University COVID-19 data.^23^ This figure compares the SARS-CoV-2 positivity rate in the study and the national SARS-CoV-2 cases reported during the data collection period.

There was, however, a correlation between the Ag-RDT positivity rate in the study and the time when testing was done. We compared SARS-CoV-2 positivity in the study and national SARS-CoV-2 cases reported during the study period (Figure 2), and there was a notable similarity between the rates of Ag-RDT positivity among study participants and national SARS-CoV-2 positivity rates.

In a crude analysis, there was also a significant association with the type of Ag-RDT used before adjusting for the confounding effect of time: 32/1,616 (2.0%) tested positive with the Abbott Panbio Ag-RDT (used at the beginning of enrollments), and 2/1,526 (0.1%) tested positive with the SD Biosensor Ag-RDT (used at the end of enrollments, from August to September 2022). However, that association disappeared when adjusted for the testing period. In the multivariable model, only the testing period and the presence of COVID-19 symptoms were independent predictors of a positive Ag-RDT test (Table 4).

Table 4 presents factors associated with SARS-CoV-2 infections. Logistic regression was used to identify the factors, both crude and adjusted for confounders.

### Polymerase chain reaction tests and SARS-CoV-2 variants among SARS-CoV-2‒positive participants.

A total of 54 samples were sent for PCR testing. Of these samples, 34 were from participants who tested positive for SARS-CoV-2 with an Ag-RDT and 20 were from participants who had COVID-19 symptoms but had a negative Ag-RDT. Of the 40 samples processed, 31 were PCR positive, whereas nine had negative PCR results. Eleven samples had SARS-CoV-2 variants identified, 22A (Omicron) in 4/11 (36%) and 22B (Omicron) in 7/11 (64%), whereas 15 PCR-positive samples had cycle threshold values >35 and sequencing could not be done.

### SARS-CoV-2 Ag-RDT service delivery and costs.

The total cost of the intervention was USD $50,446. Community mobilization was the major cost driver (26%) followed by the purchase of SARS-CoV-2 Ag-RDTs (20.5%), personnel (18%), meetings (14%), travel and transport (11%), supplies (10%), and capital costs of equipment (0.5%). The cost per individual tested was USD $15.89, and the cost per individual testing positive for SARS-CoV-2 was USD $1,484 (Supplementary Table 1 and Table 5). Table 5 present the cost of mass testing for SARS-CoV-2 using Ag-RDTs disaggregated by cost category, the cost per individual tested, and cost per individual testing positive for SARS-CoV-2.

**Table 5:** Cost per individual tested and cost per individual tested positive for SARS-CoV-2

Parameter	Value (USD)
Total Cost of Mass SARS-CoV-2 Ag-RDT	50,446
Number of Individuals Tested SARS-CoV-2	3,174
Number of Individuals Tested Positive for SARS-CoV-2	34
Cost per Individual Tested for SARS-CoV-2	15.89
Cost per Client Tested Positive for SARS-CoV-2	1,484

SARS-CoV-2 = severe acute respiratory syndrome coronavirus-2; USD = U.S. dollars.

## DISCUSSION

In this study, community SARS-CoV-2 testing using Ag-RDTs was successful in its stated goals to identify SARS-CoV-2 infections at large gathering venues among asymptomatic and symptomatic cases.

Of all individuals who turned up at the venues, about three-quarters, 78%, accepted SARS-CoV-2 testing. The majority of those who were tested were male, and the median age was 39 years. Because we did not collect demographic information of all the people who were offered testing but did not accept testing and were therefore not enrolled, we do not know if this higher proportion of middle-aged men who were tested reflects the demographics in the area or if it is due to a higher testing acceptance in this population. An acceptability study conducted in Congo-Brazzaville showed that men and those aged 30–50 years were more accepting of voluntary screening for SARS-CoV-2. In the Brazzaville study, however, acceptability for voluntary testing was 62.5%, lower than in this study (78%).^24^

In our study, refusal to test occurred at different stages: immediately after screening, during the consenting and enrollment process, and after the enrollment process. When refusals at all stages were accounted for, the refusal rate was more than 20%. The main reasons documented for refusal were participant saw no value in/no need for the test and thought they were healthy; participant disliked/felt uncomfortable with the testing procedure; and fear of the test (either fear of the pain due to the test itself or fear of the result); these results are similar to those reported from a study by McGowan et al.^25^ on SARS-CoV-2 testing acceptability and uptake. It is worth noting that responses indicating that patients did not believe they had SARS-CoV-2 infection or did not believe that their symptoms were indicative of COVID-19 may reflect the assumption that the outbreak had happened earlier and that those who were vaccinated were no longer at risk of infection.

There were also individuals who said they had come to the venue primarily to receive the SARS-CoV-2 vaccine and did not want to be tested. We believe that offering the vaccine at testing venues overall increased the SARS-CoV-2 testing coverage.

The percentage of positive SARS-CoV-2 cases detected in this study (1.07%) was higher than that obtained in the first (1.01%) and second (0.62%) rounds of mass SARS-CoV-2 Ag-RDT testing in Slovakia^26^ and among those deemed as being of moderate risk (0.8%) in a study evaluating the prevalence of SARS-CoV-2 infection in taxi stands in Johannesburg, South Africa.^27^ These differences may be due to various reasons, such as prevalence of the infection in the community (number of individuals diagnosed with SARS-CoV-2 infection), screening strategy, and sensitivity of SARS-CoV-2 Ag-RDTs. The main factor influencing positivity rate in this study was the timing of testing with regard to overall SARS-CoV-2 case notifications in the country. We started enrollments while the Omicron SARS-CoV-2 wave was still high, with more than 100 new cases notified per day, and ended them while the epidemic was stabilized, with fewer than 10 new cases notified per day.

We estimated a cost per individual tested for SARS-CoV-2 of US $15.89 and cost per individual tested positive for SARS-CoV-2 of US $1,484. To our knowledge there are no studies estimating the cost per client tested in mass screening programs in sub-Saharan Africa. However, a recent study in Germany assessing the cost of implementing Ag-RDT‒based screening programs for asymptomatic persons in high-risk settings estimated that the cost per client tested varied between US $15.63 (employing staff who performed other duties alongside SARS-CoV-2 Ag-RDTs) to US $36.56 (employing staff who worked exclusively on SARS-CoV-2 Ag-RDTs).^28^ Given that Germany is a high-income country with higher salaries, a slightly higher cost per client tested in Germany is justifiable.

We used a micro-costing approach, which was shown previously to closely reflect the real cost of an intervention^29^ and, coupled with the local SARS-CoV-2 Ag-RDT performance, confers fidelity to our results and high applicability to Kenya. Our results show that in community settings in Kenya, the SARS-CoV-2 Ag-RDT could be a useful strategy to identify asymptomatic as well as symptomatic individuals.

The price of SARS-CoV-2 Ag-RDTs corresponded to 20.5% of the total cost and influenced substantially the cost per individual tested and diagnosed with SARS-CoV-2 infection, highlighting the need to reduce the test cost burden. The implementation of community testing in the current project was USD $50,446. To maximize the gains of mass community SARS-CoV-2 Ag-RDTs, there is a need to complement it with contact tracing and to target high-risk subpopulations with an expected high yield of SARS-CoV-2 diagnoses, as suggested by López Seguí et al.^30^

Our study had some limitations. Owing to the ever-changing pandemic, we were not able to achieve the sample size planned. We started testing at the peak of a SARS-CoV-2 infection wave, which soon came to an end. People were reluctant to take the SARS-CoV-2 test, as they perceived that COVID-19 disease was no longer a problem. This also contributed to the small number of Ag-RDT‒positive cases identified. Although we had correctly estimated the positivity rate to be around 1%, with this smaller sample size, the final number of people testing positive for SARS-CoV-2 was only 34. This did not allow for a very robust analysis of the factors associated with SARS-CoV-2 infection in these communities. Although fitting a multivariable model of the factors associated with test positivity, only the time of testing or, more specifically, its correlation with the overall SARS-CoV-2 positivity rate in the country was significant. It is important to note that before adjusting for time, there was also a difference in positivity rate among the tests used, being 2% and 0.1% for the Abbott Panbio and SD Biosensor tests, respectively. The Abbott Panbio test was used from May to July when the Omicron wave was still high, whereas the SD Biosensor test was used in August and September when prevalence was lower. Because the entirety of Kiambu County was using the same batch of Abbott Panbio tests that expired in early August 2022, the change in test kits affected the whole county. We saw the same epidemic trend with very few cases after August 2022 in other areas of Kiambu where this implementation did not take place and in Kenya overall (based on the national number of new cases reported),^23^ but we cannot say if it was a true reflection of the epidemic or if this could also be due to a potential lack of sensitivity of the second test. The SD Biosensor test is known to have a lower sensitivity (sensitivity 54.1–71%, specificity 97.3–99.6%)^31^ than the Abbott Panbio test (sensitivity 85.5–86.8%, specificity 99.9–100%).^32^ We recognize that the use of different tests at different periods may have biased our estimation of case detection. In the context of community testing among individuals who are mostly asymptomatic, the use of RDTs with higher sensitivity is preferred.

Despite these limitations, this community mass testing approach successfully tested many individuals, mostly asymptomatic, and identified several SARS-CoV-2 infections, including in asymptomatic individuals. We believe this strategy helped to identify individuals with SARS-CoV-2 early for treatment if symptomatic and prevention of further spread if asymptomatic, which has been shown to be critical for the effectiveness of treatment and prevention quarantining.^33^ Our costing data also show that analyses of program inputs are a useful tool to identify main cost drivers, inform planning, and improve resource allocation for mass SARS-CoV-2 testing with Ag-RDT in community settings.

This study confirmed that targeted mass community testing using SARS-CoV-2 Ag-RDTs is a strategy that primarily reaches asymptomatic individuals. Testing in markets and shopping malls may be an effective way to intercept SARS-CoV-2 transmission when numbers in a county are beginning to rise, as there is a high prevalence of infection and good acceptability of testing in these venues.

We demonstrated that SARS-CoV-2 Ag-RDT testing could be included as part of regular MOH outreach activities, particularly during epidemics, and that offering SARS-CoV-2 Ag-RDT testing in combination with other activities such as SARS-CoV-2 vaccination can be an important element to improve attendance at a testing venue. Finally, to increase testing coverage, community awareness of the importance of knowing one’s SARS-CoV-2 infection status, particularly during epidemic waves, is critical.

## Supplemental Materials

10.4269/ajtmh.23-0756 Supplemental Materials
