# Supplementary material for: A Cross-Sectional Study of the Use of Antigen Rapid Diagnostic Tests for Community Identification of SARS-CoV-2 in Kenya
Source: Am J Trop Med Hyg. 2024 Nov 26;112(4 Suppl):17–25. doi: 10.4269/ajtmh.23-0756 (PMC11965722; doi:10.4269/ajtmh.23-0756)
Supplement: Supplemental Materials [file tpmd230756.SD1.pdf]

Table S1
Costs of mass SARS-CoV-2 Ag-RDT disaggregated by category

	**Personnel**	
**Institution**	**Position / Title**	**Cost (US$)**
EGPAF	Data manager	2,953
Ministry of health	Disease surveillance coordinators	1,181
	Nurses	2,333
	Lab technologists	2,333
	Data clerks	386
	Subtotal	6,234
	Total	9,186
**Supplies**		
**Category**	**Item**	**Cost (US$)**
Stationary and Printing	Clipboards, plastic folders	21
	Pens, Stamp pads + Ink	24
	Snacks	2,849
	Printing	41
	Sheet protectors and appointment cards	622
	Bags	176
	Covers	256
	Subtotal	3,989
Medical supplies and consumables	Gloves	681
	Bin liners	0
	Dust coats	61
	Masks	293
	Sanitizers	0
	Cooler box for PCR samples	20
	Viral transport media (VTM)	92
	Subtotal	1,147
	**Total**	**5,136**
	**SARS-CoV-2 Ag-RDT**	**10,316**
	**Equipment**	
**Item**	**Total cost (US$)**	**Annualized cost (US$)**
Laptop	835	182
**Travel**		
**Institution**	**Item**	**Cost (US$)**
EGPAF	Testing venues	866
	Sensibilization meetings	44
	Taxi	942
	Subtotal	1,851
Ministry of health	Disease surveillance coordinators (lunch + transport)	537
	Nurses (lunch + transport)	1,227

Meeting	Item	Cost (US$)
	Lab technologists (lunch + transport)	1,227
	Data clerks (lunch + transport)	626
	Subtotal	3,616
	**Total**	**5,467**
	**Community mobilization**	
	**Item**	**Cost (US$)**
	Rent of caravans	1,107
	Rent of tents/ chairs/ tables	3,540
	Demand generation materials (fliers, foldable burners, reflector jackets, caps)	1,854
	Airtime	1,840
	Allowance	4,842
	**Total**	**13,183**
	**Meeting**	
**Meeting**	**Item**	**Cost (US$)**
Virtual county officials meeting	Airtime	68
Sub county stakeholders meeting	Venue rent	1,086
	Transport reimbursement	434
Sub counties coordinators meeting	Venue rent	2,513
	Transport reimbursement	1,005
Sensitization meetings	Transport reimbursement	1,052
Coordination meeting	Lunch	818
	**Total**	**6,976**
	**Grand total**	**50,446**
